# Sociodemographic and Medical Risk Factors Associated With Antepartum Depression

**DOI:** 10.3389/fpubh.2018.00127

**Published:** 2018-05-02

**Authors:** Giridhara R. Babu, G. V. S. Murthy, Neeru Singh, Anita Nath, Mohanbabu Rathnaiah, Nolita Saldanha, R. Deepa, Sanjay Kinra

**Affiliations:** ^1^Indian Institute of Public Health (IIPH) Bangalore, Public Health Foundation of India and Wellcome Trust-DBT India Alliance, Bangalore, India; ^2^Indian Institute of Public Health, Public Health Foundation of India, Madhapur, Hyderabad, India; ^3^International Centre for Eye Health, Faculty of Infectious and Tropical Diseases, London School of Hygiene and Tropical Medicine, London, United Kingdom; ^4^IIPH Bangalore, Public Health Foundation of India, Bangalore, India; ^5^Psychiatry, Institute of Mental Health, University of Nottingham, Nottingham, United Kingdom; ^6^Clinical Epidemiology, London School of Hygiene and Tropical Medicine and University College London Hospital, London, United Kingdom

**Keywords:** pregnancy, mental distress, antenatal, stress, cohort, public hospital

## Abstract

**Background:**

The increasing recognition of antenatal depression is an emerging area of concern in developing countries. We conducted a study to estimate the prevalence of antenatal mental distress and its relation with sociodemographic factors, obstetric factors, and physiological wellbeing in pregnant women attending public health facilities in Bengaluru, South India.

**Methods:**

Nested within a cohort study, we assessed the mental status in 823 pregnant women in two public referral hospitals. Kessler Psychological Distress Scale (K-10 scale) was used to assess maternal depression. We collected information related to social-demographic characteristics and recent medical complaints. Descriptive statistics and odds ratios were calculated using SPSS version 20.

**Results:**

Results show that 8.7% of the women exhibited symptoms of antenatal depression. Sociodemographic characteristics, such as respondent occupation, husband education, husband’s occupation, total family income showed significance. First time pregnancy, anemia, and high blood pressure were also associated with mental distress.

**Conclusion:**

Our study has demonstrated feasibility of screening for mental health problems in public hospitals. Early detection of mental distress during pregnancy is crucial as it has a direct impact on the fetus. The public health facilities in low- and middle-income countries such as India should consider piloting and scaling up screening services for mental health conditions for pregnant women.

## Introduction

The increasing recognition of antenatal depression is an emerging area of concern. In contrast to affecting 7–20% of pregnant women in high-income countries, the estimates from lower and lower-middle-income countries report to be ranging from 5.2 to 32.9% (1). The pregnant women with symptoms suggestive of depression can have sudden exacerbation of existing medical disorders (2, 3). Poor mental health in pregnancy may lead to adverse pregnancy outcomes such as physical defects, low birth weight, emotional problems, preterm birth, and postnatal depression (4–6). Studies also attribute the association of behavioral, cognitive, and linguistic problems in children born to mothers suffering from depressive symptoms (7). Depression during pregnancy and the postpartum period is a major risk factor for suicidal ideation. There is sparse evidence regarding mortality associated with suicides during pregnancy in developing countries. However, the prevalence of antenatal suicidal ideation is reported in 6.3–14% of pregnant women from low-income households (8, 9). Therefore, screening for mental health disorders is vital during pregnancy, as it offers opportunities for early intervention to prevent adverse pregnancy outcomes. During the antenatal period, usually, greater importance is given in screening physical health and mental health of the pregnant woman tends to remain an area of neglect. Data from four low- and middle-income countries (LMICs) verifies that none of the countries have reported a dedicated maternal mental health service (10).

Previous studies in India have reported a wide-ranging prevalence of antenatal depression between 9.8 and 36.75% (11–13). The presence of depression among women presenting with medical complaints could be of significant importance. While there is sufficient evidence on the association of psychosocial and environmental risk factors; the association of antenatal depression with medical symptoms in LMICs including India is limited. An understanding of the sociodemographic and medical factors associated with the occurrence of depression during pregnancy could enhance better monitoring of women who are prone to develop a disorder such as this.

The present study was conducted with the objective of estimating the prevalence of symptoms of depression and its relation to sociodemographic and obstetric factors and physiological well-being in pregnant women attending public health facilities in Bengaluru, South India.

## Materials and Methods

The current study is nested within an existing cohort of 823 women, the detailed protocol of which has been published elsewhere (14). The field centers comprise two public health facilities in the city of Bengaluru. Each of these facilities registers approximately 80–120 pregnant women for antenatal care every month; recruitment was conducted from November 2013 to June 2015.

The recruitment was done after obtaining informed consent. A pre-designed approved questionnaire was used to record relevant sociodemographic details and obstetric history. Women were also asked to recall and report any medical symptoms experienced by them over the past month. Physiologic measurements included body mass index, blood pressure, and hemoglobin estimation.

The Kessler Psychological Distress Scale (K-10 scale) (15) (Appendix S1 in Supplementary Material), a well-structured questionnaire, was administered by trained research assistants (RAs). The K-10 scale is observed to be a reliable measure to screen for depression among pregnant women in developing countries (16). The WHO-issued English language version of the K10 questionnaire was translated into the local language of “*Kannada”* using the standardized WHO translation and back-translation protocol (WHO) and pretested before being finalized. The Cronbach’s Alpha score was 0.843 for all the items on K-10 scale. Privacy and confidentiality of the study participants were ensured during the study process. The K-10 has five psychological distress response categories ranked on a five-point scale (1. All the time, 2. Most of the time, 3. Some of the time, 4. A little of the time, 5. None of the time), with the score being the sum of these responses (details given in Appendix S1 in Supplementary Material).

The pregnant women were administered Kessler’s Psychological Distress Scale by the RAs during their antenatal visit to the hospital; the questionnaire was administered to all pregnant women irrespective of their gestational age.

The institutional review board at The Indian Institute of Public Health-H, Public Health Foundation of India has approved the protocol. All the analyses were carried out in SPSS version 20.

### Statistical Analysis

The main outcome variable, mental distress was dichotomized into a binary variable using a cutoff score from the K-10 scale; women with a cutoff score of ≥20 to the K10 scale are reported to be having symptoms of mental distress. Bivariate analysis of the association of sociodemographic characteristics, obstetric history, and medical symptoms with mental distress was evaluated by chi-square or fisher exact test (for cell values less than 5). Logistic regression was conducted to estimate the association between different factors and mental distress adjusting for known and unknown confounders. All those variables with a *p*-value < 0.25 on bivariate analysis were entered in the logistic regression model, even those variables with a *p*-value > 0.25 but which were known to have significant associations in previous studies were included (age, abortion, parity). More traditional levels such as 0.05 can fail in identifying variables known to be important (17, 18).

## Results

### Prevalence of Symptoms of Mental Distress

The sociodemographic characteristics of respondents with and without mental distress are displayed in Table 1. Gestational age and total household income were found to be significant and had small effect size.

**Table 1:** Sociodemographic factors and mental distress in pregnancy (*N* = 823).

Background characteristics	Mental distress		*p*-Value	Effect	Degrees of Freedom
	Present (*N* = 72)	Absent (*N* = 751)			
**Gestational age**					
First trimester (less than 12 week)	2 (11.8)	15 (88.2)	**0.001**	0.134	2
Second trimester (12–28 week)	52 (12.3)	370 (87.7)			
Third trimester (greater than 28 week)	18 (4.7)	366 (95.3)			

**Age**					
18–25	52 (9.5)	495 (90.5)	0.279	0.038	1
>25	20 (7.2)	256 (92.8)			

**Religion**					
Hindu	42 (7.7)	500 (92.3)	0.159	0.049	1
Non-Hindu	30 (11.3)	251 (88.7)			

**Respondent’s education**					
Illiterate	7 (10.0)	63 (90.0)	0.366	0.049	2
School	19 (11.2)	150 (88.8)			
College	46 (7.9)	538 (92.0)			

**Husband’s education**					
Illiterate	18 (13.2)	118 (86.8)	0.249	0.072	3
Middle school	1 (5.9)	16 (94.1)			
High school	13 (7.3)	164 (92.7)			
College	40 (8.1)	453 (91.7)			

**Subject’s occupation**					
Unemployed	64 (8.3)	706 (91.7)	0.124	0.059	1
Working	8 (15.1)	45 (84.9)			

**Husband’s Occupation**					
Unemployed	2 (25.0)	6 (75.0)	0.132	0.066	2
Unskilled worker	35 (7.7)	418 (92.3)			
Skilled worker	35 (9.7)	327 (90.3)			

**Total household income (in rupees)**					
≤6000	29 (13.2)	190 (86.8)	**0.047**	0.098	3
7000–10,000	17 (6.3)	251 (93.7)			
11,000–15,000	13 (8.1)	148 (91.9)			
>15,000	13 (7.4)	162 (92.6)			

*Statisttically significant results are in bold*.

Table 2 shows the obstetric and medical characteristics of respondents with and without mental distress. Anemia and past month medical symptoms like nausea, vomiting, cough/cold, tiredness, abdominal pain, heartburn, giddiness, Ear pain, headache, and backache were found to be significant. BMI of respondent, parity, abortion, and blood pressure did not show any significance.

**Table 2:** Obstetric cum medical factors and mental distress in pregnancy (*N* = 823).

Background characteristics	Mental distress		*p*-Value	Effect	Degrees of freedom
	Present (*N* = 72)	Absent (*N* = 751)			
**BMI**					
Normal weight	32 (9.9)	401 (92.6)	0.23	0.057	2
Under weight	5 (13.5)	32 (86.5)			
Over weight	35 (9.9)	318 (90.1)			

**Blood pressure^a^**					
High blood pressure	2 (3.7)	52 (6.3)	0.218	0.047	1
Normal blood pressure	70 (9.1)	699 (90.9)			

**Anemia**					
Anemic (Hb < 11)	33 (6.8)	455 (93.2)	**0.017**	0.085	1
Non-anemic	39 (11.6)	296 (88.4)			

**Gravida**					
Primigravida	26 (7.9)	302 (92.1)	0.531	0.024	1
Multigravida	46 (9.3)	449 (90.5)			

**Ever had an abortion**					
Yes	17 (11.2)	134 (88.8)	0.263	0.042	1
No	55 (8.2)	617 (91.8)			

**Past month medical symptoms**					
Nausea	14 (25.5)	41 (74.5)	**>0.001**	0.158	1
Vomiting	36 (19.1)	152 (80.9)	**>0.001**	0.200	1
Cough/cold	32 (13.9)	198 (86.1)	**>0.001**	0.114	1
Giddiness	38 (18.4)	168 (81.6)	**>0.001**	0.198	1
Tiredness	49 (12.3)	349 (87.7)	**>0.001**	0.122	1
Abdominal pain	27 (14.6)	158 (85.4)	**0.002**	0.111	1
Ear pain	7 (29.2)	17 (70.8)	**0.003**	0.125	1
Heartburn	19 (20.7)	73 (79.3)	**0.001**	0.149	1
Constipation	7 (21.9)	25 (78.1)	**0.017**	0.093	1
Headache	31 (16.5)	157 (83.5)	**>0.001**	0.149	1
Backache	35 (11.4)	271 (88.6)	**>0.04**	0.073	1

*Statisttically significant results are in bold*.

*^a^High Blood pressure is Systolic BP of >130 mmHg and diastolic BP of >80 mmHg*.

In Table 3, the sociodemographic factors of women were adjusted for age, gestational age, total income, respondent education, occupation, and husband’s occupation. Women in third trimester were more likely to be stressed (OR 2.67, CI 0.55–12.9) than women in early or second trimester. Women who were older than 25 years had greater risks for having mental distress than women younger than 25 years (OR 1.34, CI 0.78–2.30). Unemployed women seemed to have more mental distress than working women (OR 2.27, CI 0.98–5.26). However, the association was statistically not significant. Similarly respondents with unemployed husbands or unskilled workers had higher odds (1.34 CI 0.81–2.24) than women with husbands who were skilled workers or professionals. Women with household income less than Rs. 10,000 ($150) were at higher risk of mental distress compared to women with household income greater than Rs. 10,000.

**Table 3:** Multivariate analysis of sociodemographic risk factors and mental distress in pregnancy.

Factor	Unadjusted odds ratio (95% CI)	Adjusted odds ratio (95% CI)
**Trimester**		
I Trimester	1	1
II Trimester	0.94 (0.21–4.26)	1.04 (0.22–4.83)
III Trimester	2.71 (0.57–12.76)	2.67 (0.55–12.9)
Age (older than 25 vs. less than 25 years)	1.34 (0.78–2.30)	0.73 (0.42–1.27)
Religion: Hindu vs. non-Hindu	1.42 (0.87–2.32)	1.57 (0.93–2.64)
Respondent’s education (did not attend college vs. attended college)	0.70 (0.42–1.16)	0.81 (0.46–1.42)
Respondent’s occupation (unemployed vs. working)	1.96 (0.88–4.34)	2.27 (0.98–5.26)
Husband’s Occupation (unemployed and unskilled vs. skilled worker)	1.22 (0.75–1.99)	1.34 (0.81–2.24)

**Total income (in rupees)**		
>15,000	1	1
10,001–15,000	0.91 (0.41–2.03)	0.88 (0.39–2.00)
6001–10,000	1.18 (0.56–2.50)	1.02 (0.47–2.20)
≤6000	0.52 (0.26–1.04)	0.49 (0.23–1.04)

In Table 4, women’s medical and obstetric factors have been adjusted for age, gestational age, and parity. Anemic women had higher odds of having mental distress than non-anemic women (1.92 CI 1.17–3.15). Similarly first time mothers too had greater odds for mental distress (1.23 CI 0.73–2.07). Women with hypertension had a higher risk of mental distress than normotensive women (2.1 CI 0.49–8.93). None of the other medical symptoms reported by respondents were associated with mental distress.

**Table 4:** Multivariate analysis of medical and obstetric risk factors and mental distress in pregnancy.

Factor	Unadjusted odds ratio	Adjusted odds ratio
BMI	0.77 (0.47–1.26)	0.661 (0.40–1.08)
Hypertension	**2.60 (0.62–10.91)**	**2.1 (0.49–8.93)**
Primigravida vs. Multigravida	**1.19 (0.72–1.96)**	**1.23 (0.73–2.07)**
Anemia	**1.81 (1.11–2.95)**	**1.92 (1.17–3.15)**
Nausea	0.23 (0.12–0.46)	0.25 (0.12–0.50)
Vomiting	0.25 (0.15–0.41)	0.26 (0.16–0.46)
Cough/cold	0.44 (0.27–0.73)	0.51 (0.31–0.84)
Giddiness	0.25 (0.15–0.42)	0.28 (0.17–0.46)
Ear pain	0.21 (0.86–0.53)	0.22 (0.08–0.57)
Tiredness	0.40 (0.24–0.68)	0.47 (0.28–0.80)
Abdominal pain	0.44 (0.26–0.73)	0.50 (0.30–0.85)
Heartburn	0.30 (0.16–0.53)	0.26 (0.14–0.48)
Constipation	0.32 (0.13–0.76)	0.30 (0.12–0.75)
Headache	0.35 (0.21–0.57)	0.38 (0.23–0.64)
Backache	0.59 (0.36–0.97)	0.66 (0.40–1.09)

*Statisttically significant results are in bold*.

## Discussion

Mental health problems during the pregnancy have harmful effect on both the fetus and the mother. The results of this study suggest that symptoms of antenatal mental distress are associated with medical symptoms and other sociodemographic correlates (19). Also, there is a higher probability of depression in women post-delivery and the children are at higher risk of depression during adolescence (20). Out of 823 pregnant women, 8.7% reported having symptoms of mental distress, indicating that the antenatal depression poses a significant health issue in the study population. Previous studies in India have reported a prevalence of antenatal depression to be 9.8–36.75% (10–13). However, systematic reviews of prevalence of depression in pregnancy across the globe range from 6 to 25% (21, 22).

We found higher mental distress among women in their third trimester. This concurs with earlier evidence suggestive of progressive increase in the plasma concentration of total and free cortisol, peaking during the third trimester (23–25). Unemployed women were at a higher risk of mental distress as compared to working women. Similarly women with employed husbands appeared to have significantly lesser symptoms of mental distress compared to the women with husbands with no employment. Studies have shown excessive risk of preterm delivery in unemployed pregnant women (26). Earlier studies indicate that the fetal growth restriction (SGA) is associated with employment status of parents (27). Also, our results concur with studies reporting higher symptoms of mental distress in respondents whose total family monthly income was considerably lower (28–31).

First time mothers were more prone to mental distress in our study population; this resonates with the findings of several other studies (32, 33). It is possible that the fears associated with delivery such as losing control, something being wrong with the baby or painful contractions are more common in nulliparous women (34). However, Saisto and Halmesmäki suggest that fear of childbirth is common in nulliparous as well as in parous women. They further explain that the woman’s personal characteristics, mainly general anxiety, low self-esteem, depression, dissatisfaction with their partnership, and lack of support are important factors irrespective of parity (35).

Women with anemia had higher risk of mental distress than other women with normal hemoglobin levels. Nevertheless, few studies do not support the association between anemia and depression (36, 37). Women with higher blood pressure (systolic >130 mmHg and diastolic >80 mmHg) were at greater risk of mental distress; however, there may be temporal ambiguity of this association. Leeners et al. found emotional stress during pregnancy was associated with a 1.6-fold increased risk for hypertensive diseases in pregnancy (38).

We did not find any significant links between mental distress and any pregnancy loss. This is in concurrence with other studies, a review of articles from 1980 through 2008 to evaluate risk factors for antepartum depressive symptoms did not show obstetric history (i.e., spontaneous abortions, elective abortions, and fetal deaths *in utero*) to be significantly associated with depressive symptoms in bivariate or multivariate analyses (39).

Postpartum depression results in poorer cognitive development (40) and behavioral/emotional problems in infants (5). The main limitations of the study were the use of the Kessler’s scale as the measure of mental distress symptoms rather than a clinical diagnosis. The second limitation is to not have a homogenous population of pregnant women in terms of gestational age; the study included pregnant women from 14 week of pregnancy until 38 weeks. We believe that a longitudinal analysis of risk factors across different stages of pregnancy is required.

Early detection of mental distress during pregnancy is crucial as it has a direct impact on the fetus (41). Our study has demonstrated feasibility of screening for mental health problems in public hospitals; the pregnant women can be screened in antenatal clinics. Evidence suggests that educational interventions comprising of counseling can be successful in decreased adverse effects in pregnant women. Considering the scalability, the patient counseling by nurses and trainings for doctors can be done at all primary health centers. In addition, auxiliary nurse midwives/accredited social health activist can also be trained and skilled to assist the pregnant women about the need of screening for mental health problems and treatment. The health workers can also circulate information, education, and communication materials and motivate people as a regular activity and contribute toward efficient, empathetic management of mental health problems in pregnant women.

## Conclusion

Mental status of pregnant women play critical role in pregnancy and birth outcomes. The public health facilities in LMICs such as India should consider piloting and scaling up screening services for mental health conditions for pregnant women. Women with mental distress must be referred for further diagnostic assessment and therapy.

## Ethics Approval and Consent to Participate

The study was reviewed and approved by the institutional ethical review board (IEC) at Bangalore campus of IIPH-H. Only participants willing to participate voluntarily and those who have provided written informed consent are enrolled.

## Consent for Publication

Informed consent has been obtained from all the enrolled participants of the study.

## Availability of Data and Material

Requests for the datasets generated during and/or analyzed during the current study can be directed to the corresponding author. The Institutional ethics committee reviewing at IIPH-Bangalore will decide on providing the data.

## Ethics Statement

This study was carried out in accordance with the recommendations of “IEC Guidelines, Institutional Ethics Committee, PHFI” with written informed consent from all subjects. All subjects gave written informed consent in accordance with the Declaration of Helsinki. The protocol was approved by the “Institutional ethical Committee (IEC) at Bangalore campus of IIPH-H.”

## Author Contributions

GB has conceptualized, written, and has taken the lead in completing manuscript through all stages of preparation and submission till publication and is the first author. GM has reviewed the application from the conceptualization stage and has contributed to each version of the manuscript. AN has written and reviewed the study paper and contributed to the manuscript. MR has reviewed the application from the conceptualization stage and has contributed to the manuscript. NSingh and NSaldanha were involved in writing and analyzing the pilot data. RD was involved in writing and revising the manuscript. SK has reviewed the application from the conceptualization stage and has contributed to the manuscript. All authors have contributed to the article critically for important intellectual content and final approval of the version to be published.

## Conflict of Interest Statement

The authors declare that the research was conducted in the absence of any commercial or financial relationships that could be construed as a potential conflict of interest.

## References

[1] Fisher J Mello MCd Patel V Rahman A Tran T Holton S Prevalence and determinants of common perinatal mental disorders in women in low- and lower-middle-income countries: a systematic review. Bull World Health Organ (2012) 90(2):139–49. 10.2471/BLT.11.091850 22423165 PMC3302553

[2] Morylowska-Topolska J Makara-Studzińska M Kotarski J The influence of sociodemografic and medical variables on severity of anxiety and depressive symptoms during particular trimesters of pregnancy. Psychiatr Pol (2014) 48(1):173–86. 24946443

[3] Bisetegn TA Mihretie G Muche T. Prevalence and predictors of depression among pregnant women in Debretabor town, northwest Ethiopia. PLoS One (2016) 11(9):e0161108. 10.1371/journal.pone.0161108 27618181 PMC5019395

[4] DiPietro JA Hilton SC Hawkins M Costigan KA Pressman EK. Maternal stress and affect influence fetal neurobehavioral development. Dev Psychol (2002) 38(5):659. 10.1037/0012-1649.38.5.659 12220045

[5] O’Connor TG Heron J Glover V Team AS. Antenatal anxiety predicts child behavioral/emotional problems independently of postnatal depression. J Am Acad Child Adolesc Psychiatry (2002) 41(12):1470–7. 10.1097/00004583-200212000-00019 12447034

[6] Latendresse G Wong B Dyer J Wilson B Baksh L Hogue C. Duration of maternal stress and depression: predictors of newborn admission to neonatal intensive care unit and postpartum depression. Nurs Res (2015) 64(5):331–41. 10.1097/NNR.0000000000000117 26325275

[7] Leadbeater BJ Bishop SJ. Predictors of behavior problems in preschool children of inner-city Afro-American and Puerto Rican adolescent mothers. Child Dev (1994) 65(2):638–48. 10.2307/1131406 8013244

[8] Huang H Faisal-Cury A Chan Y-F Tabb K Katon W Menezes PR. Suicidal ideation during pregnancy: prevalence and associated factors among low-income women in São paulo, Brazil. Arch Womens Ment Health (2012) 15(2):135–8. 10.1007/s00737-012-0263-5 22382280 PMC3727148

[9] Gausia K Fisher C Ali M Oosthuizen J. Antenatal depression and suicidal ideation among rural Bangladeshi women: a community-based study. Arch Womens Ment Health (2009) 12(5):351. 10.1007/s00737-009-0080-7 19468825

[10] Bavle AD Chandahalli AS Phatak AS Rangaiah N Kuthandahalli SM Nagendra PN. Antenatal depression in a tertiary care hospital. Indian J Psychol Med (2016) 38(1):31–5. 10.4103/0253-7176.175101 27011399 PMC4782441

[11] George C Lalitha AR Antony A Kumar AV Jacob K. Antenatal depression in coastal South India: prevalence and risk factors in the community. Int J Soc Psychiatry (2016) 62(2):141–7. 10.1177/0020764015607919 26443716

[12] Hegde SS Pai KK Abhishekh HA Sandeep KR Prevalence of antenatal depression and gender preference: a cross sectional study among Mangalore population, Karnataka, India. J Pharm Biomed Sci (2013) 30(30):1011–4.

[13] Ajinkya S Jadhav PR Srivastava NN. Depression during pregnancy: prevalence and obstetric risk factors among pregnant women attending a tertiary care hospital in Navi Mumbai. Ind Psychiatry J (2013) 22(1):37. 10.4103/0972-6748.123615 24459372 PMC3895310

[14] Babu GR Garadi L Murthy G Kinra S. Effect of hyperglycaemia in pregnancy on adiposity in their infants in India: a protocol of a multicentre cohort study. BMJ Open (2014) 4(6):e005417. 10.1136/bmjopen-2014-005417 24972608 PMC4078779

[15] Kessler RC Keller MB Wittchen H-U The epidemiology of generalized anxiety disorder. Psychiatr Clin North Am (2001) 24(1):19–39. 10.1016/S0193-953X(05)70204-5 11225507

[16] Spies G Stein D Roos A Faure S Mostert J Seedat S Validity of the Kessler 10 (K-10) in detecting DSM-IV defined mood and anxiety disorders among pregnant women. Arch Womens Ment Health (2009) 12(2):69–74. 10.1007/s00737-009-0050-0 19238521

[17] Bendel RB Afifi AA Comparison of stopping rules in forward “stepwise” regression. J Am Stat Assoc (1977) 72(357):46–53. 10.1080/01621459.1977.10479905

[18] Mickey RM Greenland S. The impact of confounder selection criteria on effect estimation. Am J Epidemiol (1989) 129(1):125–37. 10.1093/oxfordjournals.aje.a115101 2910056

[19] Patel V Prince M Maternal psychological morbidity and low birth weight in India. Br J Psychiatry (2006) 188(3):284–5. 10.1192/bjp.bp.105.012096 16507972

[20] Pawlby S Hay DF Sharp D Waters CS O’Keane V. Antenatal depression predicts depression in adolescent offspring: prospective longitudinal community-based study. J Affect Disord (2009) 113(3):236–43. 10.1016/j.jad.2008.05.018 18602698

[21] Ryan D Milis L Misri N Depression during pregnancy. Can Fam Physician (2005) 51(8):1087–93. 16121830 PMC1479513

[22] Gavin NI Gaynes BN Lohr KN Meltzer-Brody S Gartlehner G Swinson T Perinatal depression: a systematic review of prevalence and incidence. Obstet Gynecol (2005) 106(5, Pt 1):1071–83. 10.1097/01.AOG.0000183597.31630.db 16260528

[23] Rosenthal HE Slaunwhite WR Jr Sandberg AA Transcortin: a corticosteroid-binding protein of plasma. X. Cortisol and progesterone interplay and unbound levels of these steroids in pregnancy. J Clin Endocrinol Metab (1969) 29(3):352–67. 10.1210/jcem-29-3-352 5812975

[24] Abou-Samra AB Pugeat M Dechaud H Nachury L Bouchareb B Fevre-Montange M Increased plasma concentration of N-terminal beta-lipotrophin and unbound cortisol during pregnancy. Clin Endocrinol (1984) 20(2):221–8. 10.1111/j.1365-2265.1984.tb00077.x 6713691

[25] Helbig A Kaasen A Malt UF Haugen G. Does antenatal maternal psychological distress affect placental circulation in the third trimester? PLoS One (2013) 8(2):e57071. 10.1371/journal.pone.0057071 23437312 PMC3577751

[26] Hanke W Saurel-cubizolles MJ Sobala W Kalinka J. Employment status of pregnant women in central Poland and the risk of preterm delivery and small-for-gestational-age infants. Eur J Pub Health (2001) 11(1):23–8. 10.1093/eurpub/11.1.23 11276567

[27] Raatikainen K Heiskanen N Heinonen S. Does unemployment in family affect pregnancy outcome in conditions of high quality maternity care? BMC Public Health (2006) 6(1):46. 10.1186/1471-2458-6-46 16504118 PMC1402277

[28] Bolton H Hughes P Turton P Sedgwick P. Incidence and demographic correlates of depressive symptoms during pregnancy in an inner London population. J Psychosom Obstet Gynaecol (1998) 19(4):202–9. 10.3109/01674829809025698 9929846

[29] Ban L Gibson JE West J Fiaschi L Oates MR Tata LJ. Impact of socioeconomic deprivation on maternal perinatal mental illnesses presenting to UK general practice. Br J Gen Pract (2012) 62(603):e671–8. 10.3399/bjgp12X656801 23265226 PMC3459774

[30] Goyal D Gay C Lee KA How much does low socioeconomic status increase the risk of prenatal and postpartum depressive symptoms in first time mothers? Womens Health Issues (2010) 20(2):96–104. 10.1016/j.whi.2009.11.003 20133153 PMC2835803

[31] Maselko J Bates L Bhalotra S Gallis JA O’Donnell K Sikander S Socioeconomic status indicators and common mental disorders: evidence from a study of prenatal depression in Pakistan. SSM Popul Health (2018) 4:1–9. 10.1016/j.ssmph.2017.10.004 29349268 PMC5769091

[32] Spice K Jones SL Hadjistavropoulos HD Kowalyk K Stewart SH. Prenatal fear of childbirth and anxiety sensitivity. J Psychosom Obstet Gynaecol (2009) 30(3):168–74. 10.1080/01674820902950538 19591052

[33] Madhavanprabhakaran GK D’Souza MS Nairy KS Prevalence of pregnancy anxiety and associated factors. Int J Afr Nurs Sci (2015) 3:1–7. 10.1016/j.ijans.2015.06.002

[34] Lowe NK. Self-efficacy for labor and childbirth fears in nulliparous pregnant women. J Psychosom Obstet Gynaecol (2000) 21(4):219–24. 10.3109/01674820009085591 11191169

[35] Saisto T Halmesmäki E. Fear of childbirth: a neglected dilemma. Acta Obstet Gynecol Scand (2003) 82(3):201–8. 10.1034/j.1600-0412.2003.00114.x 12694113

[36] Paterson JA Davis J Gregory M Holt SJ Pachulski A Stamford DE A study on the effects of low haemoglobin on postnatal women. Midwifery (1994) 10(2):77–86. 10.1016/S0266-6138(05)80249-9 8057960

[37] Patel V Kirkwood BR Weiss H Pednekar S Fernandes J Pereira B Chronic fatigue in developing countries: population based survey of women in India. BMJ (2005) 330(7501):1190. 10.1136/bmj.38442.636181.E0 15870118 PMC558019

[38] Leeners B Neumaier-Wagner P Kuse S Stiller R Rath W. Emotional stress and the risk to develop hypertensive diseases in pregnancy. Hypertens Pregnancy (2007) 26(2):211–26. 10.1080/10641950701274870 17469011

[39] Lancaster CA Gold KJ Flynn HA Yoo H Marcus SM Davis MM. Risk factors for depressive symptoms during pregnancy: a systematic review. Am J Obstet Gynecol (2010) 202(1):5–14. 10.1016/j.ajog.2009.09.007 20096252 PMC2919747

[40] Smith-Nielsen J Tharner A Krogh MT Vaever MS. Effects of maternal postpartum depression in a well-resourced sample: early concurrent and long-term effects on infant cognitive, language, and motor development. Scand J Psychol (2016) 57(6):571–83. 10.1111/sjop.12321 27611177

[41] Heron J O’Connor TG Evans J Golding J Glover V Team AS. The course of anxiety and depression through pregnancy and the postpartum in a community sample. J Affect Disord (2004) 80(1):65–73. 10.1016/j.jad.2003.08.004 15094259

