# Supplementary material for: Sociodemographic and Medical Risk Factors Associated With Antepartum Depression
Source: Front Public Health. 2018 May 2;6:127. doi: 10.3389/fpubh.2018.00127 (PMC5941824; doi:10.3389/fpubh.2018.00127)
Supplement: Supplementary file 1 [file Data_Sheet_1.PDF]

KESSLER'S PSYCHOLOGICAL DISTRESS SCALE

These questions concern how you have been feeling over the past 30 days. Tick a box below each question that best represents how you have been

1. During the last 30 days, about how often did you feel tired out for no good reason?				
1. None of the time	2. A little of the time	3. Some of the time	4. Most of the time	5. All of the time

2. During the last 30 days, about how often did you feel nervous?				
1. None of the time	2. A little of the time	3. Some of the time	4. Most of the time	5. All of the time

3. During the last 30 days, about how often did you feel so nervous that nothing could calm you down?				
1. None of the time	2. A little of the time	3. Some of the time	4. Most of the time	5. All of the time

4. During the last 30 days, about how often did you feel hopeless?				
1. None of the time	2. A little of the time	3. Some of the time	4. Most of the time	5. All of the time

5. During the last 30 days, about how often did you feel restless or fidgety?				
1. None of the time	2. A little of the time	3. Some of the time	4. Most of the time	5. All of the time

6. During the last 30 days, about how often did you feel so restless you could not sit still?				
1. None of the time	2. A little of the time	3. Some of the time	4. Most of the time	5. All of the time

7. During the last 30 days, about how often did you feel depressed?				
1. None of the time	2. A little of the time	3. Some of the time	4. Most of the time	5. All of the time

8. During the last 30 days, about how often did you feel that everything was an effort?				
1. None of the time	2. A little of the time	3. Some of the time	4. Most of the time	5. All of the time

9. During the last 30 days, about how often did you feel so sad that nothing could cheer you up?				
1. None of the time	2. A little of the time	3. Some of the time	4. Most of the time	5. All of the time

10. During the last 30 days, about how often did you feel worthless?				
1. None of the time	2. A little of the time	3. Some of the time	4. Most of the time	5. All of the time
