# Supplementary material for: Sociodemographic and Medical Risk Factors Associated With Antepartum Depression
Source: Front Public Health. 2018 May 2;6:127. doi: 10.3389/fpubh.2018.00127 (PMC5941824; doi:10.3389/fpubh.2018.00127)
Supplement: Supplementary file 2 [file Data_Sheet_2.PDF]

Appendix-2: Baseline questionnaire

INTERVIEW INFORMATION

Respondent number... ☐☐☐☐☐

Hospital Name and Code ____________________ ☐☐

District ____________________ ☐☐

Taluka____________________ ☐☐

City/Town/Village____________________

Date of interview: DAY ☐☐ MONTH ☐☐ YEAR ☐☐☐☐

Name and code of interviewer:____________________ ☐☐

Interview Place: (HOSPITAL=1, HOME=2, OTHERS=3) ____________________ ☐

(SPECIFY,IF OTHERS)

INTERVIEWER VISITS

	1	2	3	FINAL VISIT
DATE<br><br>RESULT*	________<br><br>☐	________<br><br>☐	________<br><br>☐	DAY............. ☐☐<br>MONTH......... ☐☐<br>YEAR............ ☐☐<br>RESULT CODE* ☐
NEXT VISIT: DATE<br><br>TIME	________<br><br>________	________<br><br>________		TOTAL NUMBER<br>OF VISITS ☐

***RESULT CODES:**

INTERVIEW COMPLETED=1

INTERVIEW PARTIALLY COMPLETED=2

RESPONDENT REFUSED TO TAKE PART=3

RESPONDENT SICK=4

GENERAL QUESTIONNAIRE

SOCIO-ECONOMICS STATUS

3.14 Religion	☐ Hinduism<br>☐ Christianity<br>☐ Islam<br>☐ Others, **Specify**:_____________________

3.15 Is your husband a blood relative?	☐ YES ☐ NO

3.16 **If yes to 3.15,** please specify the relation	

3.17 Number of adults in the household	☐☐

3.18 Number of children in the household	☐☐

3.19 Type of family	☐ Nuclear<br>☐ Extended<br>☐ Joint

3.20 Education of the subject and her husband	**SUBJECT**<br>☐ Illiterate<br>☐ Middle school (6-8th std)<br>☐ High school (9-10th std)<br>☐ P.U.C/ Diploma<br>☐ Graduate<br>☐ Post graduate	**HUSBAND**<br>☐ Illiterate<br>☐ Middle school (6-8th std)<br>☐ High school (9-10th std)<br>☐ P.U.C/ Diploma<br>☐ Graduate<br>☐ Post graduate

3.21 At the start of the pregnancy, was the subject working for pay?	☐ YES ☐ NO

3.22 Is the subject **currently** working for pay?	☐ YES ☐ NO	
3.23 Occupation of the subject and her husband	**SUBJECT**<br>☐ Unemployed<br>☐ Unskilled Worker<br>☐ Skilled Worker<br>☐ Petty business, shop owner<br>☐ Secretarial staff, primary school teacher	**HUSBAND**<br>☐ Unemployed<br>☐ Unskilled Worker<br>☐ Skilled Worker<br>☐ Petty business, shop owner<br>☐ Secretarial staff, primary school teacher

	☐ Semi-professional, high school teacher<br>☐ Professional	☐ Semi-professional, high school teacher<br>☐ Professional

3.24 **If yes to 3.22,** monthly income of the **subject RUPEES**	☐☐☐☐☐☐

3.25 Monthly income of the **subject's husband**<br>**RUPEES**	☐☐☐☐☐☐

3.26 Total number of **earning members** in the household	☐☐

3.27 Total monthly income of the **household**<br>**RUPEES**	☐☐☐☐☐☐

Standard of Living Index

3.28 Type of house **(CHOOSE ONLY 1)**	☐ Pucca<br>☐ Semi-pucca<br>☐ Kuccha

3.29 Does this household own this house or any other house?	☐ YES ☐ NO

3.30 How much agricultural land does this household **own**? **(CHOOSE ONLY 1)**	☐ ≥ 5 acres<br>☐ 2.0 – 4.9 acres<br>☐ < 2 acres<br>☐ None

3.31 Out of this land, how much is **irrigated**?	☐ Some<br>☐ None<br>☐ Don't know

3.32 Does this household own any livestock?	☐ YES ☐ NO

3.33 Does this household have a separate kitchen?	☐ YES ☐ NO

3.34 What type of fuel does the household **mainly** use for cooking? **(CHOOSE ONLY 1)**	☐ Wood<br>☐ Crop residues<br>☐ Dung cakes<br>☐ Coal/coke/lignite<br>☐ Charcoal<br>☐ Kerosene<br>☐ Electricity<br>☐ Liquid petroleum<br>☐ Gas<br>☐ Bio-gas
3.35 What is the **main** source of lighting for the household? **(CHOOSE ONLY 1)**	☐ Electricity<br>☐ Kerosene<br>☐ Gas<br>☐ Oil

3.36 What is the **main** source of drinking water for members of the household? **(CHOOSE ONLY 1)**	**PIPED WATER**<br>☐ Piped into Residence/Yard/Plot<br>☐ Public Tap<br>**GROUND WATER**<br>☐ Hand pump in residence/Yard/Plot<br>☐ Public Hand pump<br>**WELL WATER (WELL IN RESIDENCE/YARD/PLOT)**<br>☐ Covered well<br>☐ Open well<br>☐ Public Well<br>**SURFACE WATER**<br>☐ Spring<br>☐ River/Stream<br>☐ Pond/Lake<br>☐ Dam<br>☐ Rain water<br>☐ Tanker truck
3.37 What kind of **toilet** facility does the household have? **(CHOOSE ONLY 1)**	**FLUSH TOILET**<br>☐ Own Flush Toilet<br>☐ Shared Flush Toilet<br>☐ Public Flush Toilet

	**PIT TOILET/ LATRINE**<br>☐ Own pit toilet/latrine<br>☐ Shared toilet/latrine<br>☐ Public toilet/latrine<br>☐ NO FACILITY/ BUSH/ FIELD

3.38 Does this household own any of the following?	A Mattress	☐ YES	☐ NO
	A Pressure Cooker	☐ YES	☐ NO
	A Chair	☐ YES	☐ NO
	A Cot or Bed	☐ YES	☐ NO
	A Table	☐ YES	☐ NO
	A clock or watch	☐ YES	☐ NO
	An electric fan	☐ YES	☐ NO
	A bicycle	☐ YES	☐ NO
	A radio or transistor	☐ YES	☐ NO
	A sewing machine	☐ YES	☐ NO
	A telephone	☐ YES	☐ NO
	A mobile phone	☐ YES	☐ NO
	A refrigerator	☐ YES	☐ NO
	A color television	☐ YES	☐ NO
	A moped, scooter or motorcycle	☐ YES	☐ NO
	A auto	☐ YES	☐ NO
	A car	☐ YES	☐ NO
	A tempo	☐ YES	☐ NO
	A water pump	☐ YES	☐ NO
	A bullock cart	☐ YES	☐ NO
	A thresher	☐ YES	☐ NO
	A tractor	☐ YES	☐ NO

MEDICAL AND OBSTETRICS HISTORY

4.4 Obstetrics history

4.4a Gravida	☐ 0<br>☐ 1<br>☐ 2<br>☐ 3<br>☐ 4<br>☐ 5
4.4b Parity<br>(Number of times a woman has given birth to a fetus with a gestational age of 28 weeks or more, regardless of whether the child was born alive or was stillborn)	☐ 0<br>☐ 1<br>☐ 2<br>☐ 3<br>☐ 4
4.4c Abortion<br>(Number of times a woman has given birth to a fetus with a gestational age of 28 weeks or less)	☐ 0<br>☐ 1<br>☐ 2<br>☐ 3<br>☐ 4<br>☐ 5
4.4d Living Children<br>(Number of times a woman has given birth to a live fetus)	☐ 0<br>☐ 1<br>☐ 2<br>☐ 3<br>☐ 4

MONTHLY MEDICAL HISTORY

7.16 Since pregnancy/ past one month**,** have you experienced the following symptoms or diseases?

Read the following symptoms or diseases and mark out all symptoms/diseases that the SUBJECT has experienced.

**Symptoms / Disease**	YES	NO	DK
a) Diarrhea (>3 loose, watery stools /day)	☐	☐	☐
b) Dysentery (Diarrhea containing mucus and blood in feces)	☐	☐	☐
c) Nausea	☐	☐	☐
d) Vomiting	☐	☐	☐
e) Fever	☐	☐	☐
f) Productive Cough / cold	☐	☐	☐

g) Sore throat	☐	☐	☐
h) Wheezing (high-pitched whistling sound during breathing)	☐	☐	☐
i) Giddiness	☐	☐	☐
j)Tiredness	☐	☐	☐
k) Abdominal pain	☐	☐	☐
l) Ear pain	☐	☐	☐
m) Heart burn	☐	☐	☐
n) Constipation	☐	☐	☐
o) Vaginal bleeding	☐	☐	☐
p) Pain during urination	☐	☐	☐
q) Convulsions	☐	☐	☐
r) Mouth ulcers	☐	☐	☐
s) Headache	☐	☐	☐
t) Backache	☐	☐	☐
u) Skin related problems **specify** ___________________________	☐	☐	☐
w) Others, **Specify** _________________________________	☐	☐	☐

BLOOD PRESSURE OF THE MOTHER

7.19 Blood pressure
Systolic Clinical reading ☐☐☐ mm/Hg

7.20 Blood pressure
Diastolic Clinical reading ☐☐☐ mm/Hg

7.21 Blood pressure
Systolic Automated reading ☐☐☐ mm/Hg

7.22 Blood pressure
Diastolic Automated reading ☐☐☐ mm/Hg

MOTHER ANTHROPOMETRY

Measurements	Reading 1	Reading 2	Reading 3
8.7ba Weight (kg)	☐☐☐ . ☐	☐☐☐ . ☐	☐☐☐ . ☐
8.7bb Height (cm)	☐☐☐ . ☐	☐☐☐ . ☐	☐☐☐ . ☐

BIOCHEMICAL INVESTIGATION

Date of blood test ________________________	
a. Blood group	☐ A Positive ☐ A Negative<br>☐ B Positive ☐ B Negative<br>☐ AB Positive ☐ AB Negative<br>☐ O Negative ☐ O Positive ☐ DK/NA<br>☐ RH Negative ☐ RH Positive ☐ DK/NA
b. Hemoglobin (g/dl)	☐☐.☐ DK/NA ☐
